# The clinical course, biochemical markers, and clinical outcomes of COVID-19 positive patients from the third wave in Pakistan: A retrospective cohort study

**DOI:** 10.1016/j.amsu.2022.103599

**Published:** 2022-04-19

**Authors:** Muhammad Tanveer Alam, Asad Mehdi, Yumna Timsaal, Muhammad Rehan, Arjun Kumar, Imran Sarwar Shaikh, Farah Yasmin, Gul Muhammad Memon, Nisar Ahmed, Muhammad Sohaib Asghar

**Affiliations:** a Department of Internal Medicine, Dow University of Health Sciences, Karachi, Pakistan; b Department of Internal Medicine, Civil Hospital, Sukkur, Pakistan; c Department of Internal Medicine, Liaquat National Hospital and Medical College, Karachi, Pakistan

**Keywords:** COVID-19, SARS-CoV-2, Infection, Pandemic, Severity

## Abstract

**Background:**

Third wave of COVID-19 has affected several countries. Case fatality rates from first and second waves are expected to be surpassed by the current wave due to various variant transmissions. This study was aimed to compare and contrast the significant clinical markers between survivors and non-survivors during the third wave of COVID-19 to assess severity and prognosis.

**Methods:**

It includes all the patients who were diagnosed with COVID-19 polymerase chain reaction (PCR) during the third wave, and were monitored for their disease course and outcomes. A total of 209 patients were included in the analysis via non-probability consecutive sampling method.

**Results:**

The median age was higher in non-surviving patients (p = 0.010). Majority of deaths occurred in intensive care patients (p < 0.001) and those with diabetes (p = 0.032) and hypertension (p = 0.003). Fever was the most predominant symptom in all patients (78.9%), dyspnea was common among expired individuals (p = 0.043) while recovered patients were more likely to be asymptomatic (p = 0.044). Gastrointestinal symptoms were not found marked during this wave. Being on ventilator has higher mortality (p < 0.001). Predominant radiological findings were interstitial patches or infiltrate (43.7%). Multivariable analysis showed hypertension (p = 0.042), BiPAP/CPAP (p < 0.001), being on ventilator (p = 0.004), and ARDS (p < 0.001) was associated with poor survival while patchy interstitial infiltrates on X-ray had good survival probability (p = 0.032). On Kaplan-Meier survival analysis, hypertension (p = 0.003), BiPAP/CPAP (p = 0.008), ventilator (p = 0.025), ICU stay (p = 0.001), high-grade fever (p = 0.001), and ARDS (p < 0.001) had reduced cumulative survival.

**Conclusion:**

Certain biochemical markers were more predictive of disease severity in the third-wave than the preceding waves.

## Highlights

• This study was aimed to compare the significant clinical markers between survivors and non-survivors during the third wave of COVID-19.
• Certain biomarkers and patients' factors were more predictive of disease severity in the third-wave than the preceding waves.

## 1 Introduction

The coronavirus disease 2019 (COVID-19) caused by the severe acute respiratory syndrome coronavirus 2 (SARS-CoV-2) that originated in Wuhan, Hubei Province has been a major cause of global public health concern [1]. It has affected approximately 264 million lives worldwide, as of December 2021 inclusive of five million deaths, with the virus having an estimated reproductive number of 2.87 (95%CI: 2.39–3.44) [2]. Due to its multifaceted clinical prognosis, insidious onset and non-specific disease course, COVID-19 patients continue to receive delayed care as a result of which the development of acute respiratory distress syndrome (ARDS) has been reported, severe enough to require respiratory support [3]. 33% of infected patients are found to have asymptomatic illness while those who present with symptoms can range from mild to critical, with a higher frequency of mild disease state [4,5]. The case fatality rate shows an increase from <0.6% to 2.2% for patients over 60 years old and rises to over 9.3% at age 80, proving to be highly age dependent [6]. After surviving the first and second wave of COVID-19, the third wave hit various countries like Spain in early 2021 exhibiting various differential features such as greater number of reinfections and household contacts, a highly transmissible B.1.1.7 strain, less severe cases, and lower mortality rates [7,8]. A study done in Japan showed that the most common underlying comorbidities reported during the third wave were diabetes, hypertension, and pre-existing pulmonary disease [9]. Inflammatory biomarkers also played a key role as for every 1 mg/dL rise in C-reactive protein (CRP), 10 IU/L rise in lactate dehydrogenase (LDH), and 100 ng/mL rise in ferritin, the risk for moderate to severe disease was seen to surge by approximately 18%, 13%, and 9%, respectively [9].

As the positivity rate in Pakistan surged to >10%, the country witnessed its third wave of COVID-19 in March 2021 [10]. The third wave hit Pakistan in the spring season similar to the first wave, indicating the possibility of pollen to enhance disease transmission [11]. This study was aimed to compare and contrast the significant clinical markers between survivors and non-survivors during the third wave of COVID-19 in Karachi, Pakistan to assess severity and prognosis.

## 2 Material and methods

It was conducted in a single-center, retrospectively designed observational study, during the months of March till May 2021, when the third wave was at peak in the region. The center serves as one of largest facility for COVID-19 isolation and intensive care in the city of Karachi. The study includes all the patients who were diagnosed with COVID-19 polymerase chain reaction (PCR) positive via either nasopharyngeal or oropharyngeal swab. The diagnostic kit exploits the principle of real-time fluorescence (RT-PCR) with USA-WA1/2020 stock concentration 2.8E+05 TCID50/mL and a lower detection limit of 0.003 TCID50/mL. The patients were monitored for their disease course and outcomes. A total of 209 patients were included in the analysis, out of which one-third were in intensive care unit (ICU), and 60 patients died during the hospital stay (28.7%). The exclusion criteria were patients ongoing treatment within the hospital at the time study recruitment (n = 28), patients with incomplete data on chart review (n = 47), and those who were discharged early for home isolation without complete recovery since no follow-up was available for them in chart review (n = 61).

The manuscript conformed to STROCCS guidelines for reporting cohort studies [12]. The research protocol was registered with registry board of Dow University Hospital (UIN# IRB/DUH/2021/053). Owing to retrospective data collection, informed consent was waived. The statistical analysis was conducted by the Statistical Package for the Social Sciences (SPSS version 25.0, IBM Corp., Armonk, NY). Descriptive variables were presented as median and interquartile range and were then compared using both non-parametric Mann-Whitney *U* test. The comparison of categorical data (presented as frequency and percentage) was performed either using the Chi-square test or Fisher's exact test as appropriately indicated. A p-value of <0.05 was considered statistically significant (2-tailed). A receiver operating characteristic (ROC) analysis was also obtained to determine the predictive laboratory parameters for outcome as death. For categorical variables, univariate and multivariate regression was performed to associate their outcome as death. Kaplan-Meier survival curves were illustrated to demonstrate the survival logs among the study variables and cumulative survival of the study subjects.

## 3 Results

The median age was higher in non-surviving patients (p = 0.010), with no gender discrimination (p = 0.793). Time since diagnosis (p = 0.036) and time spent in hospital (p = 0.005) were both shorter in non-surviving patients. Majority of deaths occurred in intensive care patients (p < 0.001) and those with diabetes (p = 0.032) and hypertension (p = 0.003) as shown in Table 1. Fever was the most predominant symptom in all the patients (78.9%) as well as in expired patients (p = 0.035). Dyspnea was also found common among expired individuals (p = 0.043) while recovered patients were more likely to be asymptomatic (p = 0.044). With increasing grade of fever, the risk of mortality increases (p < 0.001). Such as >103 ° F temperature has a mortality rate up to 56% as opposed to 28% in 101–103 °F and 20% in <100 ° F. Gastrointestinal symptoms were not found marked during this wave. Being on ventilator or use of BiPAP/CPAP has higher mortality (p < 0.001) as opposed to increased recovery with oxygen mask (p < 0.001) and nasal canula (p = 0.009). Predominant radiological findings were interstitial patches or infiltrate (43.7%) but with good recovery rate (p = 0.001) in contrast to worse outcomes with ARDS (p < 0.001) as shown in Table 2.

**Table 1:** Baseline data of the study population (n = 209).

S.no	Table 1: Baseline data of the study population (n = 209).				p-value
#	Variables	Total (n = 209)	Recovered (n = 149)	Expired (n = 60)	
1	Median age (IQR)	56.00 (50.00–65.00)	56.00 (46.00–63.00)	60.00 (52.00–70.00)	0.010^†^
2	Male gender	149 (71.3%)	107 (71.8%)	42 (28.2%)	0.793*
	Female gender	60 (28.7%)	42 (70.0%)	18 (30.0%)	
3	Time since diagnosis (in days)	14.00 (10.00–17.50)	14.00 (10.00–18.00)	12.00 (8.75–15.00)	0.036^†^
	Time since hospitalization	8.00 (5.00–11.00)	8.00 (5.00–12.00)	6.50 (3.00–9.25)	0.005^†^
4	ICU stay	67 (32.1%)	24 (35.8%)	43 (64.2%)	<0.001*
	Non-ICU stay	142 (67.9%)	125 (88.0%)	17 (12.0%)	
5	Diabetes	84 (40.2%)	53 (63.1%)	31 (36.9%)	0.032*
	Hypertension	105 (50.2%)	65 (61.9%)	40 (38.1%)	0.003*
	COPD	13 (6.3%)	9 (69.2%)	4 (30.8%)	0.884*
	CKD	25 (12.1%)	15 (60.0%)	10 (40.0%)	0.195*
	CAD	27 (13.0%)	17 (63.0%)	10 (37.0%)	0.323*
	CLD	12 (5.8%)	9 (75.0%)	3 (25.0%)	0.754*
	Asthma	10 (4.8%)	8 (80.0%)	2 (20.0%)	0.527*

Data presented as either median (IQR), or frequency (percentage).

* indicates either Chi-square test or Fisher's exact test used to compute the p-value.^†^*indicates Mann Whitney U test to compute the p-value*.

IQR: interquartile range; ICU: intensive care unit; COPD: chronic obstructive pulmonary disease; CKD: chronic kidney disease; CAD: coronary artery disease; CLD: chronic liver disease.

**Table 2:** Clinical profiles, symptomatology, and radiological findings of the study population (n = 209).

Characteristics	Variables	Frequency (%)	Recovered (n = 149)	Expired (n = 60)	p-value
Symptomatology	Fever	165 (78.9)	112 (67.9)	53 (32.1)	0.035*
	Dry cough	124 (59.6)	89 (71.8)	35 (28.2)	0.783
	Cough with sputum	37 (17.7)	22 (59.5)	15 (40.5)	0.079
	Sore throat	31 (14.8)	24 (77.4)	7 (22.6)	0.414
	Chest pain	18 (8.6)	10 (55.6)	8 (44.4)	0.123
	Dyspnea	131 (62.7)	87 (66.4)	44 (33.6)	0.043*
	Fatigue	75 (35.9)	51 (68.0)	24 (32.0)	0.431
	Rhinitis	16 (7.7)	10 (62.5)	6 (37.5)	0.419
	Headache	15 (7.2)	8 (53.3)	7 (46.7)	0.111
	Arthralgia/Myalgia	31 (14.9)	21 (67.7)	10 (32.3)	0.602
	Vomiting	11 (5.3)	7 (63.6)	4 (36.4)	0.564
	Nausea	18 (8.6)	11 (61.1)	7 (38.9)	0.318
	Diarrhea	14 (6.7)	11 (78.6)	3 (21.4)	0.533
	Abdominal pain	10 (4.8)	5 (50.0)	5 (50.0)	0.127
	Asymptomatic	16 (7.7)	15 (93.8)	1 (6.2)	0.044*
Grading of fever (n = 165)	99-100 °F	29 (17.6)	23 (79.3)	6 (20.7)	0.304
	101-102 °F	104 (63.0)	75 (72.1)	29 (27.9)	0.793
	>103 °F	32 (19.4)	14 (43.8)	18 (56.3)	<0.001*
Mode of respiratory support	Ventilator (invasive)	26 (12.4)	3 (11.5)	23 (88.5)	<0.001*
	BiPAP/CPAP	44 (21.0)	20 (45.5)	24 (54.5)	<0.001*
	Oxygen by mask	90 (43.1)	79 (87.8)	11 (12.2)	<0.001*
	High flow nasal canula	27 (12.9)	25 (92.6)	2 (7.4)	0.009*
	None	22 (10.5)	22 (100.0)	0 (0.0)	0.002*
Chest X-ray	Normal	29 (13.9)	29 (100.0)	0 (0.0)	<0.001*
	Consolidation	19 (9.1)	15 (78.9)	4 (21.1)	0.439
	Ground glass opacities	9 (4.3)	7 (77.8)	2 (22.2)	0.660
	Nodular opacity	28 (13.4)	16 (57.1)	12 (42.9)	0.075
	Pleural effusion	3 (1.4)	1 (33.3)	2 (66.7)	0.143
	ARDS	30 (14.4)	5 (16.7)	25 (83.3)	<0.001*
	Interstitial patchy infiltrates	91 (43.5)	76 (83.5)	15 (15.5)	0.001*
Zonal predominance	Upper zone	6 (2.9)	5 (83.3)	1 (16.7)	0.508
	Middle zone	116 (55.5)	77 (66.4)	39 (33.6)	0.080
	Lower zone	87 (41.6)	67 (77.0)	20 (23.0)	0.123
Location of patch	Central	93 (44.5)	71 (76.3)	22 (23.7)	0.148
	Peripheral	112 (53.6)	76 (67.9)	36 (32.1)	0.238
	Both	4 (1.9)	2 (50.0)	2 (50.0)	0.342

Data presented as n (%)/Frequency (%). All p-values calculated by either Chi-square test or Fisher's exact test. * indicates significant p-value of less 0.05 (two-tailed).

ARDS: acute respiratory distress syndrome; BiPAP: bilevel positive airway pressure CPAP: continuous positive airway pressure; F: Fahrenheit; n: number of subjects.

All vital markers were extreme in non-surviving group except pulse rate (p = 0.089). Higher TLC (p < 0.001), neutrophils (p = 0.008), MCV (p = 0.008) and APTT (p = 0.007) were noticed in non-survivors among the hematological indices along with lower lymphocytes (p = 0.004). Deranged urea (p < 0.001), creatinine (p < 0.001), chloride (p = 0.036), potassium (p = 0.032), bicarbonate (p < 0.001), and magnesium (p = 0.020) were found significant among renal profile and electrolytes panel. All the inflammatory biomarkers including CRP, ferritin, LDH, procalcitonin and D-dimer were markedly increased in non-survived patients (p < 0.001), while liver function enzymes were not discriminative among either group as shown in Table 3. Multivariable analysis showed Hypertension (p = 0.042), BiPAP/CPAP (p < 0.001), being on ventilator (p = 0.004), and ARDS (p < 0.001) would be associated with poor survival while patchy interstitial infiltrates on X-ray has good survival probability (p = 0.032) as shown in Table 4.

**Table 3:** Comparison of vital markers and admitting laboratory investigations among the outcome of patients (n = 209).

Variables	Recovered (n = 149)	Expired (n = 60)	p-value
Vital signs on admission			
Pulse (per min)	108.00 (98.00–112.00)	99.00 (90.25–112.75)	0.089
Systolic blood pressure (mmHg)	130.00 (121.00–139.00)	113.00 (110.00–120.00)	<0.001^a^
Diastolic blood pressure (mmHg)	80.00 (80.00–89.00)	80.00 (78.00–80.00)	0.001^a^
Respiratory rate (per min)	25.00 (21.00–29.00)	31.00 (29.00–34.00)	<0.001^a^
Oxygen saturation (%)	92.00 (89.00–96.00)	85.50 (82.00–88.00)	<0.001^a^
Hematological profile			
Leukocytes ( × 10^9^per L)	10.08 (6.59–14.48)	15.40 (8.23–21.24)	<0.001^a^
Hemoglobin (g/L)	12.34 (10.80–13.42)	11.95 (9.90–13.80)	0.576
Lymphocytes (%)	16.00 (10.00–22.00)	10.00 (5.75–16.00)	0.004^a^
Neutrophils (%)	80.00 (72.00–86.00)	85.00 (76.00–90.00)	0.008^a^
Monocytes (%)	4.00 (3.00–6.00)	4.00 (2.00–6.00)	0.515
Eosinophils (%)	2.00 (1.00–2.00)	1.50 (0.00–2.50)	0.460
Basophils (%)	1.00 (1.00–1.00)	0.00 (0.00–1.00)	0.063
Platelets ( × 10^9^per L)	218.00 (157.00–271.00)	204.00 (138.75–322.25)	0.733
Mean corpuscular volume (fL)	85.00 (80.00–88.66)	87.00 (84.24–90.00)	0.008^a^
Prothrombin time (seconds)	11.00 (10.35–11.85)	11.30 (10.40–11.90)	0.271
Activated partial thromboplastin time (seconds)	25.00 (22.85–27.50)	29.00 (25.32–36.92)	0.007^a^
International normalized ratio	1.00 (1.00–1.10)	1.10 (1.00–1.17)	0.059
Biochemistry panel			
Urea (mg/dL)	34.00 (23.00–53.00)	65.00 (31.00–123.00)	<0.001^a^
Creatinine (mg/dL)	1.00 (0.78–1.50)	1.64 (0.98–3.38)	<0.001^a^
Sodium (mEq/L)	137.00 (134.00–140.00)	136.00 (129.00–140.00)	0.076
Potassium (mEq/L)	3.90 (3.60–4.30)	4.20 (3.60–5.40)	0.032^a^
Chloride (mEq/L)	102.00 (99.00–105.00)	101.00 (96.00–105.00)	0.036^a^
Bicarbonate (mEq/L)	22.00 (20.00–24.00)	19.00 (16.00–22.00)	<0.001^a^
Calcium (mg/dL)	8.54 (7.89–9.02)	8.28 (7.63–8.82)	0.224
Magnesium (mg/dL)	2.07 (1.86–2.34)	2.35 (1.93–2.58)	0.020^a^
Phosphate (mg/dL)	3.00 (2.45–3.79)	3.23 (2.50–5.92)	0.055
Inflammatory biomarkers			
C-reactive protein (mg/dL)	13.88 (3.44–21.90)	24.37 (15.67–36.00)	<0.001^a^
Ferritin (ng/mL)	748.50 (380.50–1379.75)	1611.00 (809.00–3269.00)	<0.001^a^
Procalcitonin (ng/ml)	0.28 (0.09–0.93)	1.88 (0.50–5.34)	<0.001^a^
Lactate dehydrogenase (U/L)	413.50 (312.00–528.75)	815.00 (560.00–1164.00)	<0.001^a^
D-dimer (mcg/mL)	1.30 (0.66–3.73)	3.92 (0.97–9.78)	<0.001^a^
Liver function enzymes			
Alanine aminotransferase (U/L)	37.00 (21.00–65.00)	36.00 (24.00–55.00)	0.931
Aspartate aminotransferase (U/L)	45.50 (30.50–68.25)	52.00 (35.50–109.00)	0.140
Total bilirubin (mg/dL)	0.55 (0.38–0.71)	0.59 (0.40–1.03)	0.206
Direct bilirubin (mg/dL)	0.24 (0.19–0.37)	0.32 (0.18–0.59)	0.321
Indirect bilirubin (mg/dL)	0.29 (0.22–0.44)	0.26 (0.18–0.40)	0.492
Gamma glutamyl transferase (U/L)	56.00 (34.50–102.00)	65.00 (39.50–100.00)	0.469
Alkaline phosphatase (U/L)	86.00 (69.00–123.50)	102.00 (73.50–148.00)	0.242

All p-values calculated by Mann Whitney *U* test.

a indicates significant p-value of less than 0.05 (two-tailed).

**Table 4:** Multivariable analysis of associated factors with death in COVID-19 patients (n = 209).

Variables	Odds ratio (OR)	95% confidence interval		p-value	Adjusted odds ratio (aOR)	95% confidence interval		p-value
		Lower	upper			lower	upper	
Age >50 years	2.815	1.319	6.007	0.007*	1.831	0.497	6.752	0.363
Male gender	0.916	0.475	1.768	0.793	–	–	–	–
ICU stay	4.261	1.889	9.613	<0.001*	2.998	0.950	9.464	0.061
Invasive ventilation	30.252	8.615	106.231	<0.001*	31.341	7.315	101.821	0.004*
BiPAP/CPAP	4.300	2.137	8.651	<0.001*	6.334	3.229	10.349	<0.001*
Diabetes	1.936	1.055	3.554	0.033*	1.546	0.578	4.132	0.386
Hypertension	2.585	1.381	4.838	0.003*	2.871	1.040	7.924	0.042*
CKD	1.787	0.753	4.237	0.188	–	–	–	–
COPD	1.111	0.329	3.756	0.865	–	–	–	–
Asthma	0.603	0.124	2.928	0.531	–	–	–	–
CAD	1.553	0.666	3.620	0.308	–	–	–	–
CLD	0.819	0.214	3.134	0.770	–	–	–	–
Presence of Fever	2.501	1.046	5.979	0.039*	2.134	0.207	21.953	0.524
Fever >103 °F	4.023	1.795	9.017	0.001*	2.750	0.824	9.183	0.100
Dry cough	0.944	0.513	1.735	0.852	–	–	–	–
Cough with sputum	1.924	0.919	4.030	0.083	–	–	–	–
Sore throat	0.688	0.279	1.694	0.416	–	–	–	–
Chest pain	2.138	0.800	5.714	0.130	–	–	–	–
Dyspnea	1.960	1.015	3.786	0.045*	1.449	0.417	3.368	0.159
Fatigue	1.544	0.535	4.457	0.421	–	–	–	–
Rhinitis	0.848	0.421	1.706	0.644	–	–	–	–
Headache	2.328	0.805	6.735	0.119	–	–	–	–
Arthralgia/Myalgia	1.129	0.536	2.770	0.636	–	–	–	–
Vomiting	1.449	0.408	5.143	0.566	–	–	–	–
Nausea	1.675	0.610	4.500	0.322	–	–	–	–
Diarrhea	0.660	0.178	2.455	0.536	–	–	–	–
Abdominal pain	2.618	0.729	9.397	0.140	–	–	–	–
Consolidation	0.638	0.203	2.008	0.442	–	–	–	–
Ground glass	0.700	0.141	3.468	0.662	–	–	–	–
Nodular opacity	2.078	0.917	4.709	0.080	–	–	–	–
ARDS	20.571	7.353	57.552	<0.001*	16.334	5.299	50.349	<0.001*
Interstitial patches	0.320	0.164	0.624	0.001*	0.334	0.129	0.913	0.032*
Upper zone	0.488	0.056	4.268	0.517	–	–	–	–
Middle zone	1.737	0.934	3.229	0.081	–	–	–	–
Lower zone	0.612	0.327	1.145	0.124	–	–	–	–
Central patch	0.636	0.344	1.177	0.150	–	–	–	–
Peripheral patch	1.572	0.849	2.910	0.150	–	–	–	–

* indicates significant p-value of less than 0.05 (two-tailed). Model is adjusted for age, gender and length of hospital stay.

ARDS: acute respiratory distress syndrome, ICU: intensive care unit; BiPAP: bilevel positive airway pressure CPAP: continuous positive airway pressure; F: Fahrenheit; COPD: chronic obstructive pulmonary disease; CKD: chronic kidney disease; CAD: coronary artery disease; CLD: chronic liver disease; COVID-19: coronavirus disease 2019.

**Fig. 1:**
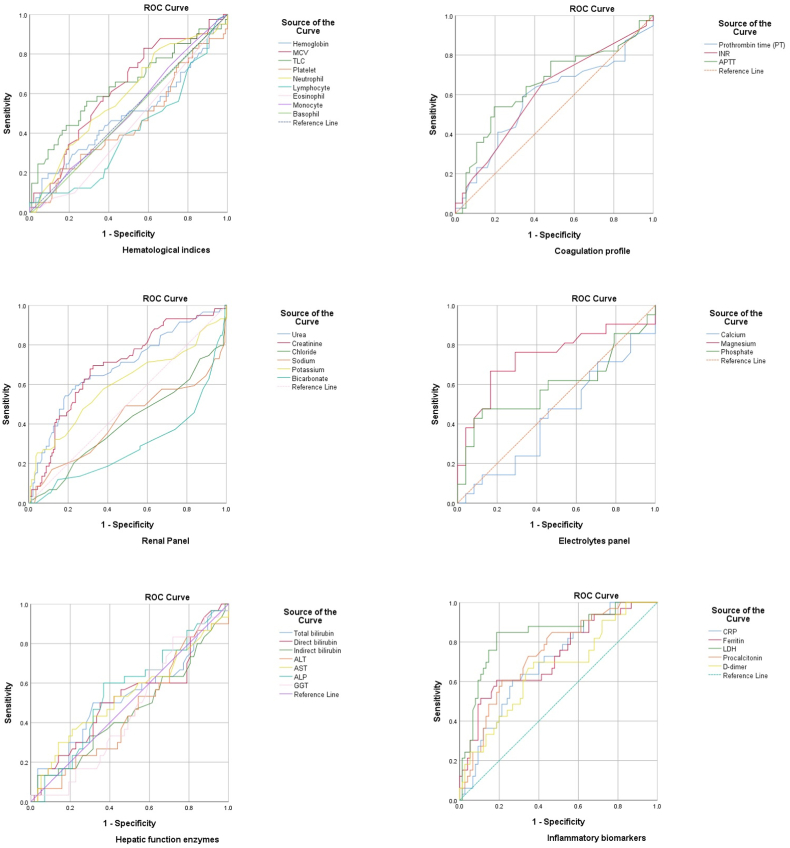
Receiver operating characteristic curves for all the laboratory markers including hematological indices, renal profile, electrolytes panel, coagulation profile, inflammatory markers and liver function enzymes.

**Fig. 2:**
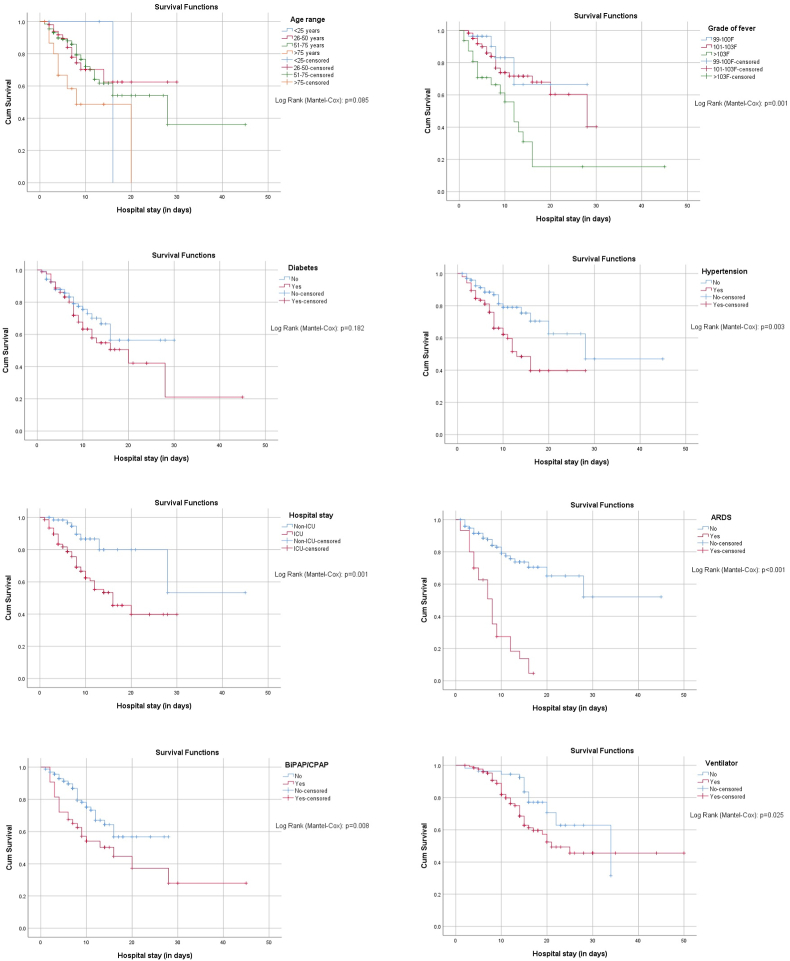
Kaplan-Meier survival logs for study variables such as age, fever grading, diabetes, hypertension, ICU stay, invasive (ventilator) and mechanical (non-invasive) ventilation, and ARDS.

On receiver operating analysis, TLC (AUC: 0.666), neutrophils (AUC: 0.618), lymphocytes (AUC: 0.369) MCV (AUC: 0.617), APTT (AUC: 0.658), serum urea (AUC: 0.688), creatinine (AUC: 0.693), chloride (AUC: 0.406), potassium (AUC: 0.596), bicarbonate (AUC: 0.287), magnesium (AUC: 0.623), CRP (AUC: 0.692), ferritin (AUC: 0.713), LDH (AUC: 0.809), procalcitonin (AUC: 0.781) and D-dimer (AUC: 0.673) were found associated with mortality as shown in Supplementary Table 5 and Fig. 1. On Kaplan-Meier survival analysis, hypertension (p = 0.003), BiPAP/CPAP (p = 0.008), ventilator (p = 0.025), ICU stay (p = 0.001), high-grade fever (p = 0.001), and ARDS (p < 0.001) was associated with reduced cumulative survival as shown in Fig. 2.

## 4 Discussion

The present study, to the best of our knowledge, is the first report comparing clinical characteristics between survivors and non-survivors during the course of the third wave of the COVID-19 pandemic in Karachi, Pakistan. In this retrospective single‐center study done in Karachi, we describe subsequent risk factors and variables to highlight the contrast in adverse outcomes of the surviving and non-surviving patients from March till May 2021. Our study concluded to have a higher age but no gender discrimination in the non-surviving group. The median age was 56 years in the recovered group and 60 years in the expired group. Previous surveys undertaken to assess disease severity showed advanced age to be a strong predictor for marked clinical deterioration due to the association of several comorbidities [[13], [14], [15], [16]]. A study done by Zhang et al. in Wuhan, China showed 50.7% of the participants to be males, further establishing no gender inclination linked to adverse disease outcome [17].

The most common complaints on admission were fever, cough and dyspnea. Nearly 78.9% of patients in our study predominantly reported fever at the onset of symptomatic illness making fever grading >103 °F an important prognostic factor between the surviving and non-surviving group. Wang, Dawei et al. also identified that the most prevalent symptoms in the emergency department (ED) for COVID cases were fever, dry cough, and dyspnea at 98.6%, 59.4% and 31.2%, respectively [18]. As suggested by another multicenter case series, only 43.8% of patients reported fever at the onset, while 87.9% reported having had a fever during the course of their hospitalization [19]. A retrospective cohort study identified an association between hyperthermia and mortality rate where mortality was seen to be lower (41%) in COVID patients that recorded a temperature of less than 102 °F compared to a higher mortality rate of nearly 70.6% and 100% in patients who had peak temperatures greater than 104 °F and 105 °F, respectively [20]. Identifying mortality predictors such as body temperature gives us a chance to better understand and manage adverse disease outcomes since there are certain predictors such as age and gender that cannot be controlled. However, it remains disputed in literature whether normothermia should be achieved in febrile intensive care unit (ICU) patients since hyperthermia boosts the immune response to evade further pathogen invasion [21,22].

We noticed that comorbidities such as diabetes, hypertension, and chronic pulmonary disease were prevalent amongst patients and resulted in a greater mortality rate. There are several factors that link these chronic conditions and poor prognosis of patients with COVID-19. For example, diabetes contributes towards patients becoming immunocompromised which puts them at a greater risk of infectivity and a slower rate of recovery, posing a higher risk of developing respiratory complications in the ICU during their hospital stay [23]. A meta-analysis that included 16,000 patients reported a significant association between mortality from COVID-19 and diabetes. The disease was found to be two times more severe in diabetic patients and diabetic patients were also two times more likely to expire from the infection; odds ratio (OR) = 1.97. These patients required invasive ventilation and also had a greater likelihood of developing ARDS [24,25]. To establish diabetes as a sole determinant of adverse outcomes in COVID-19 patients, a study was done in China that aimed to contrast clinical characteristics between diabetic and non-diabetic COVID-19 infected patients. Researchers found that those patients who only reported diabetes, in the absence of other comorbidities, were observed to be at a higher risk of experiencing severe pulmonary complications such as pneumonia. The prevalence of diabetes is responsible for initiating an uncontrolled inflammatory response, resulting in hypercoagulability and releasing enzymes from injured tissue. They also found various inflammatory serum biomarkers to be high in these patients such as CRP, D-dimers, ferritin, and IL-6 [24]. A rise in the aforementioned serum markers causes an inflammatory storm in diabetic patients which plays an influential role in aggravating COVID symptoms [9,24,26].

Another comorbid found in our study that played an impactful role in mortality from COVID-19 was hypertension. Nearly half the patients were hypertensive and 38% of hypertensive patients expired due to COVID complications. A survey found that hypertension could be 2.5 times more likely to develop a severe disease or result in a case of COVID mortality [27]. Hypertension causes disruption of physiological processes at the level of the vasculature, predisposing hypertensive individuals to a critical course of illness [28]. SARS-CoV-2 gains entrance into the cells by attaching to angiotensin-converting enzyme 2 (ACE2) receptors. Due to the massive inflammatory response occurring as a result of endothelial cell activation, there is an increased number of ACE2 receptors being anchored to the cell surface [23]. However, an intriguing conclusion drawn from several studies indicates that consumption of antihypertensive agents like ACE inhibitors and angiotensin receptor blockers could lead to a greater number of ACE2 receptors being expressed on the cell surface. Due to higher expressivity of ACE2 receptors, a larger quantity of these receptors are also available for SARS-CoV-2 to attach and subsequently infect the cells [23,27,29,30]. Discontinuation of antihypertensive agents is not advised in such individuals because these drugs often offer renal and cardiovascular protection that might be crucial for ICU support in COVID-19 patients. Due to minimal clinical evidence, there is a lack of sufficient literature supporting the benefit of suspending antihypertensive drugs in these patients [29]. Moreover, studies show that ACE2 receptors protect the lungs from developing ARDS so the benefits could outweigh the harm [31].

There are certain limitations to this study, amongst which the most prominent factors are a small cohort and single-center design. Owing to missing data for a substantial number of patients, that were excluded from the study might have led to selection bias. However, the study was able to associate many clinical and laboratory parameters with mortality.

## 5 Conclusion

Certain biomarkers and patients’ factors were more predictive of disease severity in the third-wave than the preceding waves. Factors like D-dimer levels were found to be predictive of prognosis in previous waves did not have significant correlations with disease severity in our cohort. LDH, procalcitonin, and serum ferritin levels had the most significant correlations with disease prognosis in this wave.

## Funding statement

The authors declare that they have no commercial associations (e.g. consultancies, stock ownership, equity interest, patent/licensing arrangement etc.) or funding with this article.

## Data availability statement

Data can be made available on request from corresponding author.

## Ethical approval statement

Ethical approval was taken in this study from institutional review board of Dow University Hospital (Ref:App.# IRB/DUH/2021/053), and consent to participants was not required due to retrospective nature of the study.

## Provenance and peer review

Not commissioned, externally peer reviewed.

## Author contribution

M.T.A, F.Y and M.S.A conceived the idea; G.M.M, N.A, A.M, M.S.A, and F.Y, collected the data; M.T.A, and M.S.A analyzed and interpreted the data; I.S.S, A.K, Y. T, and M.R did write up of the manuscript; and finally, F.Y, M.S.A, and M.T.A reviewed and revised the manuscript for intellectual content critically. All authors approved the final version of the manuscript.

## Provenance and peer review

Externally peer reviewed not commissioned.

## Consent

Studies on patients or volunteers require ethics committee approval and fully informed written consent which should be documented in the paper.

Authors must obtain written and signed consent to publish a case report from the patient (or, where applicable, the patient's guardian or next of kin) prior to submission. We ask Authors to confirm as part of the submission process that such consent has been obtained, and the manuscript must include a statement to this effect in a consent section at the end of the manuscript, as follows: “Written informed consent was obtained from the patient for publication of this case report and accompanying images. A copy of the written consent is available for review by the Editor-in-Chief of this journal on request”.

Patients have a right to privacy. Patients’ and volunteers' names, initials, or hospital numbers should not be used. Images of patients or volunteers should not be used unless the information is essential for scientific purposes and explicit permission has been given as part of the consent. If such consent is made subject to any conditions, the Editor in Chief must be made aware of all such conditions.

Even where consent has been given, identifying details should be omitted if they are not essential. If identifying characteristics are altered to protect anonymity, such as in genetic pedigrees, authors should provide assurance that alterations do not distort scientific meaning and editors should so note.

Consent to participate was not required due to retrospective nature of the study.

## Guarantor

The Guarantor is the one or more people who accept full responsibility for the work and/or the conduct of the study, had access to the data, and controlled the decision to publish.

## Declaration of competing interest

The authors have no conflict of interest.
