# Supplementary material for: The clinical course, biochemical markers, and clinical outcomes of COVID-19 positive patients from the third wave in Pakistan: A retrospective cohort study
Source: Ann Med Surg (Lond). 2022 Apr 19;77:103599. doi: 10.1016/j.amsu.2022.103599 (PMC9015951; doi:10.1016/j.amsu.2022.103599)
Supplement: Multimedia component 2 [file mmc2.docx]

| **Table 5: ROC statistics of the COVID-19 patients for expiry (death).** | | | | | | | | | | | | |
| --- | --- | --- | --- | --- | --- | --- | --- | --- | --- | --- | --- | --- |
| **#** | **Variables** | **Determined cut-off** | **AUC** | **S.E** | **95% C.I** | **SEN (%)** | **SPE (%)** | **PPV (%)** | **NPV (%)** | **ACC (%)** | **Y.I** | **p-value** |
| **Hematological indices** | | | | | | | | | | | | |
| **1** | **Hemoglobin** | 12.25 g/L | **0.475** | 0.047 | 0.383–0.567 | 48.3 | 48.2 | 28.2 | 69.0 | 48.3 | 0.956 | 0.576 |
|  | **MCV** | 85.58 fL | **0.617** | 0.043 | 0.532–0.702 | 62.7 | 57.4 | 38.1 | 78.6 | 59.0 | 1.191 | 0.009* |
|  | **TLC** | 10.49 ×10^9^/L | **0.666** | 0.045 | 0.577–0.754 | 68.3 | 52.8 | 38.0 | 79.8 | 57.4 | 1.201 | <0.001* |
|  | **Platelet** | 234.00 ×10^9^/L | **0.485** | 0.048 | 0.392–0.578 | 43.3 | 57.3 | 29.9 | 70.7 | 53.2 | 0.996 | 0.733 |
|  | **Neutrophil** | 74.50 % | **0.618** | 0.045 | 0.531–0.705 | 83.1 | 35.5 | 35.0 | 83.3 | 49.5 | 1.176 | 0.008* |
|  | **Lymphocyte** | 11.50 % | **0.369** | 0.044 | 0.283–0.455 | 56.9 | 68.6 | 42.9 | 79.3 | 65.1 | 1.245 | 0.004* |
|  | **Monocyte** | 3.50 % | **0.470** | 0.048 | 0.376–0.563 | 60.4 | 34.4 | 27.6 | 67.7 | 42.0 | 0.938 | 0.520 |
|  | **Eosinophil** | 0.50 % | **0.430** | 0.051 | 0.329–0.531 | 9.8 | 75.8 | 14.3 | 67.0 | 56.4 | 0.846 | 0.194 |
|  | **Basophil** | 0.50 % | **0.485** | 0.053 | 0.380–0.589 | 7.3 | 89.8 | 23.1 | 69.8 | 65.4 | 0.961 | 0.778 |
| **Coagulation profile** | | | | | | | | | | | | |
| **2** | **PT** | 11.15 sec | **0.559** | 0.055 | 0.452–0.666 | 57.8 | 61.2 | 44.1 | 73.2 | 60.0 | 1.180 | 0.272 |
|  | **APTT** | 28.15 sec | **0.658** | 0.058 | 0.545–0.771 | 52.5 | 80.0 | 61.8 | 73.2 | 69.5 | 1.315 | 0.007* |
|  | **INR** | 1.025 | **0.598** | 0.053 | 0.494–0.703 | 65.9 | 53.8 | 44.6 | 73.7 | 58.2 | 1.187 | 0.072 |
| **Renal and electrolytes panel** | | | | | | | | | | | | |
| **3** | **Urea** | 46.50 mg/dL | **0.688** | 0.043 | 0.603–0.772 | 64.4 | 69.1 | 46.9 | 82.1 | 67.7 | 1.325 | <0.001* |
|  | **Creatinine** | 1.16 mg/dL | **0.693** | 0.041 | 0.613–0.773 | 69.5 | 67.6 | 47.7 | 83.9 | 68.2 | 1.361 | <0.001* |
|  | **Sodium** | 137.50 mEq/L | **0.421** | 0.050 | 0.323–0.518 | 49.2 | 51.4 | 29.9 | 70.6 | 66.7 | 0.996 | 0.077 |
|  | **Potassium** | 4.05 mEq/L | **0.596** | 0.048 | 0.502–0.691 | 57.6 | 61.9 | 39.1 | 77.5 | 60.6 | 1.185 | 0.032* |
|  | **Chloride** | 104.50 mEq/L | **0.406** | 0.047 | 0.314–0.498 | 25.4 | 71.7 | 27.8 | 69.2 | 57.8 | 0.961 | 0.037* |
|  | **Bicarbonate** | 19.50 mEq/L | **0.287** | 0.043 | 0.203–0.371 | 54.2 | 83.6 | 58.2 | 81.3 | 74.8 | 1.368 | <0.001* |
|  | **Calcium** | 8.40 mg/dL | **0.401** | 0.083 | 0.239–0.563 | 47.6 | 48.1 | 41.7 | 54.2 | 47.9 | 0.947 | 0.244 |
|  | **Magnesium** | 2.18 mg/dL | **0.623** | 0.054 | 0.518–0.729 | 66.7 | 63.6 | 48.4 | 78.9 | 64.6 | 1.293 | 0.020* |
|  | **Phosphate** | 3.00 mg/dL | **0.603** | 0.054 | 0.496–0.710 | 67.4 | 52.5 | 44.9 | 73.7 | 57.9 | 1.189 | 0.055 |
| **Hepatic function enzymes** | | | | | | | | | | | | |
| **4** | **Total bilirubin** | 0.71 mg/dL | **0.575** | 0.061 | 0.455–0.695 | 47.1 | 76.3 | 45.7 | 77.2 | 67.5 | 1.224 | 0.206 |
|  | **Direct bilirubin** | 0.33 mg/dL | **0.559** | 0.062 | 0.437–0.681 | 50.0 | 68.8 | 40.5 | 76.4 | 63.1 | 1.177 | 0.322 |
|  | **Indirect bilirubin** | 0.29 mg/dL | **0.456** | 0.066 | 0.328–0.585 | 41.9 | 54.7 | 31.0 | 66.0 | 50.5 | 0.956 | 0.492 |
|  | **ALT** | 23.50 U/L | **0.505** | 0.054 | 0.399–0.611 | 81.1 | 30.5 | 31.3 | 80.6 | 44.7 | 1.106 | 0.931 |
|  | **AST** | 46.50 U/L | **0.589** | 0.062 | 0.468–0.711 | 63.6 | 51.4 | 36.8 | 76.0 | 55.1 | 1.140 | 0.141 |
|  | **γGT** | 52.00 U/L | **0.544** | 0.058 | 0.430–0.658 | 66.7 | 49.3 | 37.3 | 76.6 | 57.7 | 1.150 | 0.470 |
|  | **ALP** | 98.00 U/L | **0.571** | 0.061 | 0.451–0.690 | 60.6 | 63.8 | 41.7 | 78.7 | 53.2 | 1.234 | 0.242 |
| **Inflammatory biomarkers** | | | | | | | | | | | | |
| **5** | **CRP** | 15.56 mg/L | **0.692** | 0.043 | 0.607–0.776 | 76.5 | 53.5 | 39.4 | 85.2 | 60.0 | 1.290 | <0.001* |
|  | **LDH** | 592.50 U/L | **0.809** | 0.039 | 0.732–0.887 | 74.5 | 83.3 | 63.3 | 89.4 | 80.8 | 1.568 | <0.001* |
|  | **Ferritin** | 783.00 ng/mL | **0.713** | 0.045 | 0.625–0.801 | 77.6 | 53.1 | 38.8 | 86.1 | 59.9 | 1.297 | <0.001* |
|  | **Procalcitonin** | 1.22 ng/mL | **0.781** | 0.042 | 0.698–0.864 | 65.0 | 81.8 | 61.9 | 83.7 | 76.5 | 1.458 | <0.001* |
|  | **D-dimer** | 2.31 mcg/mL | **0.673** | 0.046 | 0.583–0.763 | 68.0 | 66.2 | 43.6 | 84.3 | 66.7 | 1.332 | <0.001* |
| * Indicates p <0.05. COVID-19: coronavirus disease 2019. SEN: sensitivity; SPE: specificity; C.I: confidence interval; ACC: accuracy; Y.I: Youden’s index; PPV: positive predictive value, NPV: negative predictive value, AUC: area under curve, S.E: standard error of mean, ROC: receiver operating characteristic; MCV: mean corpuscular volume; TLC: total leukocyte count; PT: prothrombin time; APTT: activated partial thromboplastin time; INR: international normalized ratio; ALT: alanine aminotransferase; AST: aspartate aminotransferase; γGT: gamma glutamyl transferase; ALP: alkaline phosphatase; CRP: c-reactive protein; LDH: lactate dehydrogenase. | | | | | | | | | | | | |
